# Dysregulation of the TOX-RUNX3 pathway in cutaneous T-cell lymphoma

**DOI:** 10.18632/oncotarget.5742

**Published:** 2019-05-03

**Authors:** Brittany O. Dulmage, Oleg Akilov, John R. Vu, Louis D. Falo, Larisa J. Geskin

**Affiliations:** ^1^ Department of Dermatology, University of Pittsburgh, Pittsburgh, Pennsylvania, USA; ^2^ Department of Dermatology, Columbia University, New York, New York, USA

**Keywords:** cutaneous T-cell lymphoma, Sézary syndrome, gene expression, romidepsin, TOX

## Abstract

Studies have examined gene expression changes in Sézary syndrome (SS), but disease pathogenesis remains largely unknown, and diagnosis and treatment are difficult. *TOX* is a transcription factor involved in CD4+ T-cell development with downstream effects on *RUNX3*, a known tumor suppressor gene. We sought to identify genes involved in SS disease pathogenesis with the potential to enable diagnosis and treatment. We utilized previously reported transcriptome sequencing data to construct a list of candidate genes, which was narrowed using pathway analysis. qRT-PCR confirmed *TOX* upregulation (>7 fold increase) in SS (*n* = 5), as well as two established markers, *PLS3* and *KIRD3DL2.* We also evaluated expression of members of the TOX-RUNX3 pathway and confirmed downregulation of *RUNX3* (0.59 fold decrease) and upregulation of *GATA3* (2 fold increase). Moreover, *TOX* and *RUNX3* expression were significantly inversely proportional. Using siRNA to suppress TOX, we demonstrated that TOX knockdown rescues RUNX3 expression and reduces cell viability. We evaluated TOX protein expression in paraffin-embedded skin biopsies with immunohistochemistry, showing nuclear staining of CTCL infiltrates, suggesting it is a candidate diagnostic biomarker. Further studies validating our findings and evaluating the TOX-RUNX3 pathway and the role of *TOX* as a disease marker and therapeutic target are warranted.

## INTRODUCTION

Cutaneous T-cell lymphomas (CTCL) are a heterogeneous group of T-cell lymphoproliferative disorders involving the skin. The most common form of CTCL is mycosis fungoides (MF), which accounts for half of CTCL cases and classically presents with indolent skin-limited patches, plaques, and tumors [1]; however, disease progression and extracutaneous dissemination are possible [2, 3]. Sézary syndrome (SS) is a rare leukemic variant of CTCL that has a high mortality rate and a median overall survival rate of 5.1 years, which decreases to as Sézary cell burden increases [4]. Sézary cells are neoplastic CD4+ lymphocytes found in skin, lymph nodes, and peripheral blood. Early detection of SS is associated with positive clinical outcome, but diagnosis is complicated by histological and clinical similarities between SS and other dermatoses [5].

Despite attempts to define gene expression in CTCL, the molecular biology and pathogenesis of the disease remain poorly understood. Genomic studies over the last ten years have attempted to characterize gene expression in CTCL [6]. These studies have aimed to establish biomarkers useful in diagnosis, to elucidate disease pathogenesis, and to discover targets for therapy. Several genes have been identified as upregulated in multiple studies including *PLS3*, *DNM3*, and *Twist* [7–11]. Despite the consensus on dysregulation of a small number of genes, the role of these dysregulated genes in pathogenically relevant pathways has not been well characterized. Further, little is known how the effects of treatment and clinical responses correlate with dysregulated gene expression.

Recently, we and others have identified dysregulation of the gene *TOX* and its protein product, thymocyte selection-associated high mobility group box protein (TOX) in MF [12–15]. TOX is a transcription factor that contains an HMG box DNA binding domain function necessary for the development of CD4+ cell lineages and lymphoid tissue but not typically expressed in mature circulating CD4+ cells. [16] Overexpression of TOX was also found to have prognostic implications in CTCL as it correlated with thicker lesions, such as patches and plaques as well as disease progression and mortality. [14] TOX was also upregulated in lesional skin from SS patients [15] and in SS peripheral blood mononuclear cells by microarray analysis [11].

To further investigate gene dysregulation in SS, we pooled our previously reported next generation sequence-based transcriptome data comparing CD4+ malignant T-cells with non-malignant CD4+ cells from the same SS patient [17, 18], and identified genes likely to be differentially expressed. A number of these genes were selected for validation and analysis, including *TOX*. After confirming dysregulation of *TOX*, we also sought to characterize the expression of its downstream targets and the effects of treatment on its expression.

Here, we present our results, confirming the differential expression of *TOX* and other identified genes, and we propose potential roles for a new pathway in disease pathogenesis.

## RESULTS

### Candidate gene prioritization and biomarker selection

To identify genetic signatures for SS, we utilized two different forms of transcriptome analysis, based on the results of our previously reported datasets. First, we isolated CD4+ T-cells from both SS patients and normal controls and compared gene expression between the two using next generation sequencing-based transcriptome analysis [18]. This analysis was also used to compare normal control CD4+ T-cells to lesional skin from MF patients [18]. Second, we isolated highly purified Sézary cells and matched non-malignant CD4+ T-cells from the same patient and compared gene expression in these two cell populations as we previously described [17]. The results of these two data collection methods were examined and differentially expressed genes were selected. Genes most likely to be biomarkers and oncogenes were identified with the use of Ingenuity and Metacore software. Finally, an exhaustive literature search of gene expression in CTCL was conducted and genes identified in previous publications were compared with our data. Based on the literature and software analysis, five genes were initially selected for validation: *TOX*, *PLS3*, *PDCD6*, *KIR3DL2*, and *Integrin-β1*. The expression levels of these genes using our sequence-based method as well as their biological relevance are displayed in Table 1.

**Table 1:** Genes selected for reverse transcriptase analysis.

Gene	SS to Norm	MF to Norm	Biological Relevance
T-plastin (*PLS3*)	83.00	12.00	PLS3 is an actin-building protein not normally expressed in T-cells [34]. It has a role in SS cell survival, and migration [31].
Thymocyte selection associated high mobility group box protein (*TOX*)	21.47	1.38	TOX is a transcription factor highly expressed in the thymus necessary for the development of CD4+ cells but not normally expressed in mature CD4+ cells [16].
Killer cell Ig-like receptor 3DL2 (*KIR3DL2*)	19.67	n/a	KIR3DL2 inhibits natural killer–mediated lysis after interaction with HLA-A, may prevent elimination of tumor cells [36].
Integrin β1 (*ITGB1*)	16.67	3.19	Integrins play a role in cellular shape, motility and the cell cycle [37]. In SS, ITGB1 may increase skin homing [38].
Programmed Cell Death 6 (*PDCD6*)	13	0.16	PDCD6 product participates in T-cell receptor-programmed cell death and may have a role in cancer survival pathways [39].

Ratios of overexpression comparing SS CD4+ cells to normal CD4+ cells as well as MF lesional skin to normal CD4+ cells as determined by previously reported sequence-based analysis are displayed as well as biological significance.

### Gene expression analysis and immunohistochemical staining

**Figure 1:**
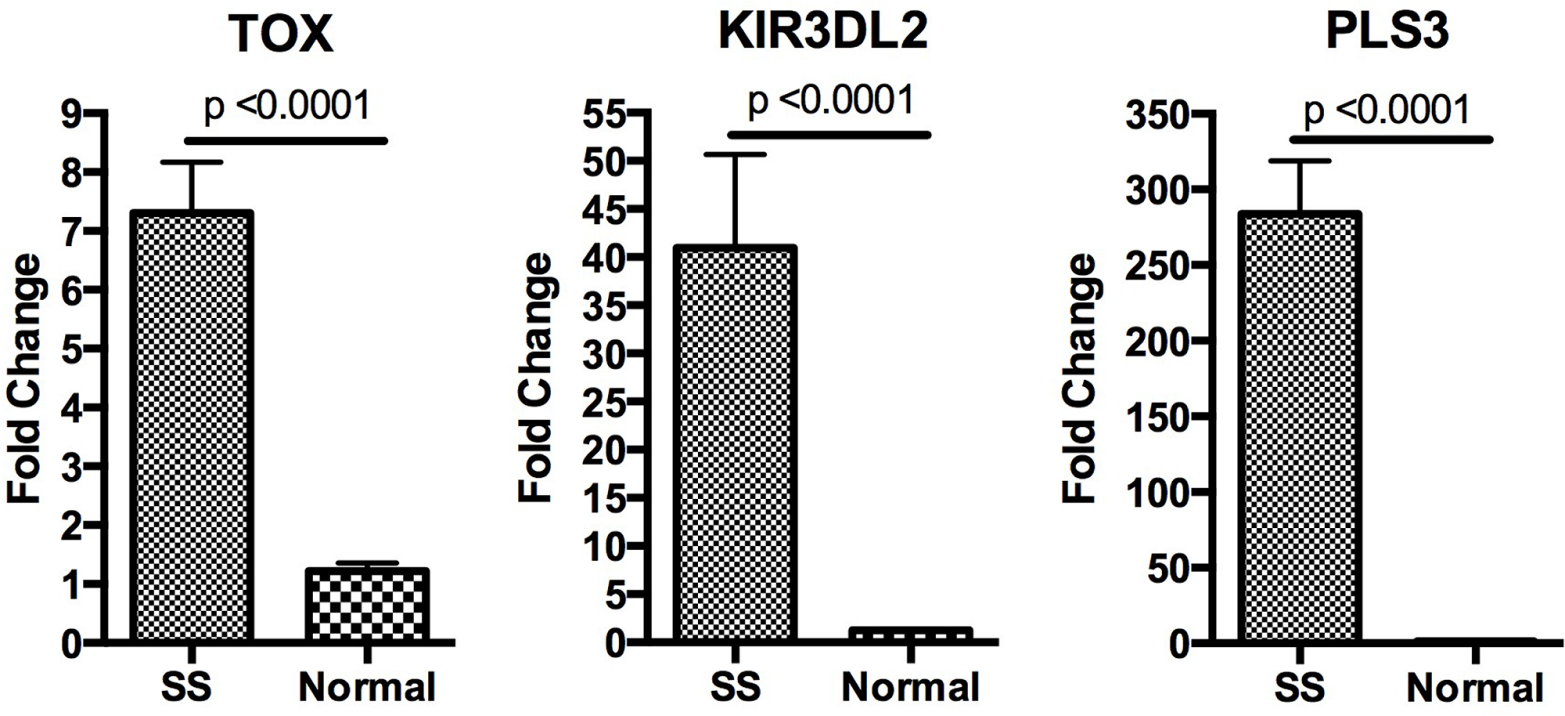
Analysis of gene expression Relative expression of *PLS3*, *TOX*, and *KIR3DL2* as measured by qRT-PCR is shown with fold change representing the average expression level in give SS patients relative to the average expression level in three normal controls. Data are represented as mean +\− SEM. Expression levels were normalized the housekeeping gene *B2M*. All three genes were upregulated in SS with *p*-values < 0.0001.

PLS3 and KIR3DL2 have been consistently reported as upregulated in SS, and their expression has been confirmed by flow cytometry [6, 19, 20]. For our initial and verifying studies, samples of purified CD4+ T cells were obtained from five SS patients (Supplemental table) and three healthy blood donors. Real-time PCR analysis confirmed statistically significant overexpression of *TOX*, *PLS3*, and *KIR3DL2* in SS CD4+ T-cells compared to healthy blood donor samples (Figure 1). *PLS3* and *KIR3DL2* were over-expressed in all five patient samples in each set of experiments. *TOX* was over-expressed in four of the five patient samples in each set of experiments. As there has been little reported on the role of TOX in CTCL, we selected it for further analysis.

**Figure 2:**
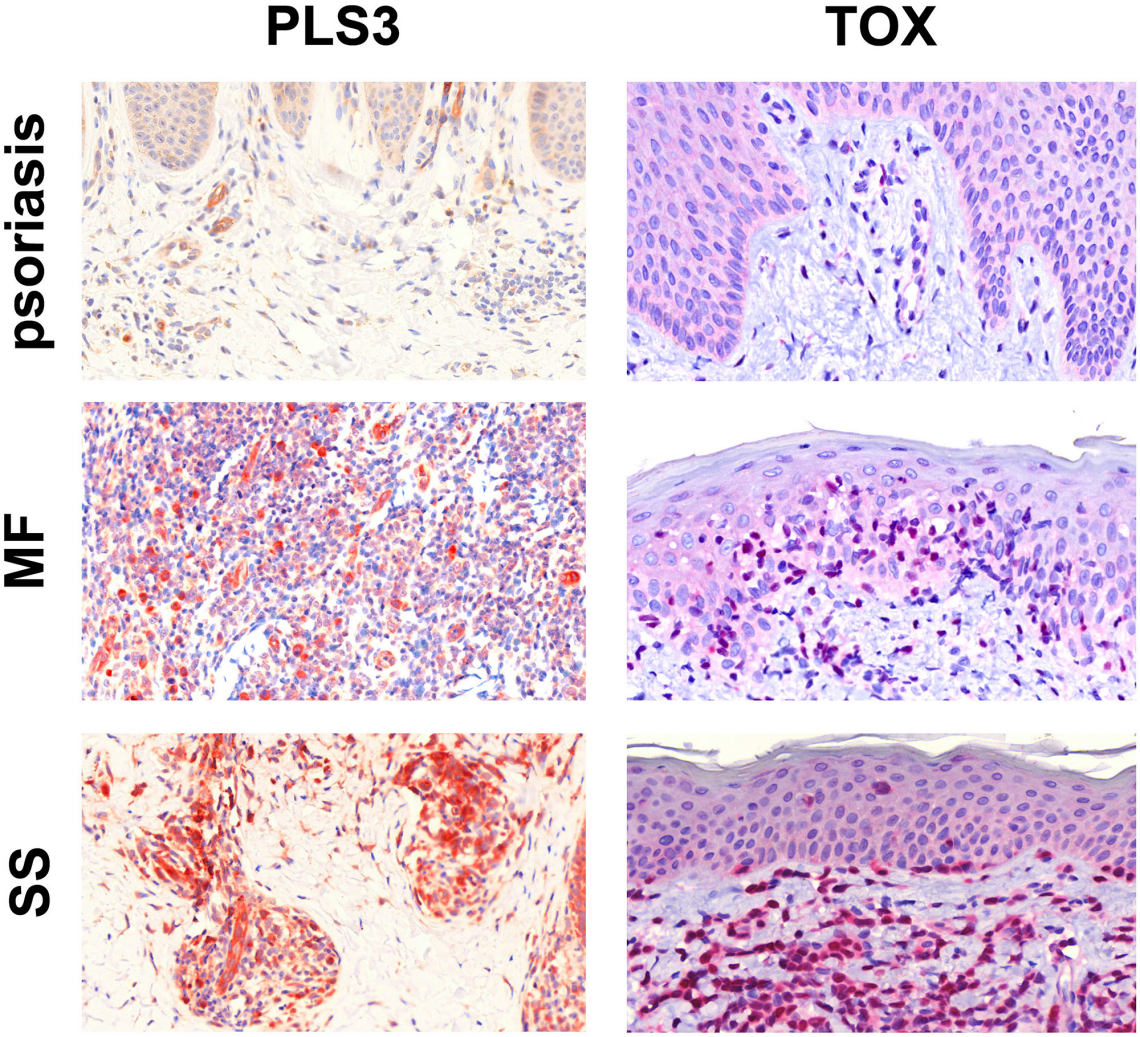
Immunohistochemical staining for PLS3 and TOX IHC staining for PLS3 of skin specimens from patients with psoriasis, MF, and SS shows strong cytoplasmic expression of PLS3 in CTCL infiltrate but not psoriasis. In all three samples, vessels are positive due to the presence of actin-bound PLS3 [35]. IHC staining for TOX shows strong, nonuniform nuclear staining in CTCL infiltrate but not psoriasis. All images are 40X and were captured using a Zeiss Mirax Midi Slide Scanner with Pannoramic MIDI/Viewer software.

Diagnosis of CTCL represents a significant challenge due to the absence of definitive disease markers. Uniquely expressed proteins are ideal candidates to serve as biomarkers of the disease, enabling its diagnosis and management. To evaluate TOX for utility as a diagnostic marker we employed immunohistochemical staining of routine tissue sections from four MF patients, two SS patients, and nine psoriasis controls using commercial antibodies chosen based on their established use in staining formalin fixed paraffin embedded tissues. As PLS3 has not been formally studied with IHC, it was included as well. Both TOX and PLS3 were detected in SS and MF samples (Figure 2).

### Treatment with romidepsin, but not pralatrexate, alters expression of selected genes

**Figure 3:**
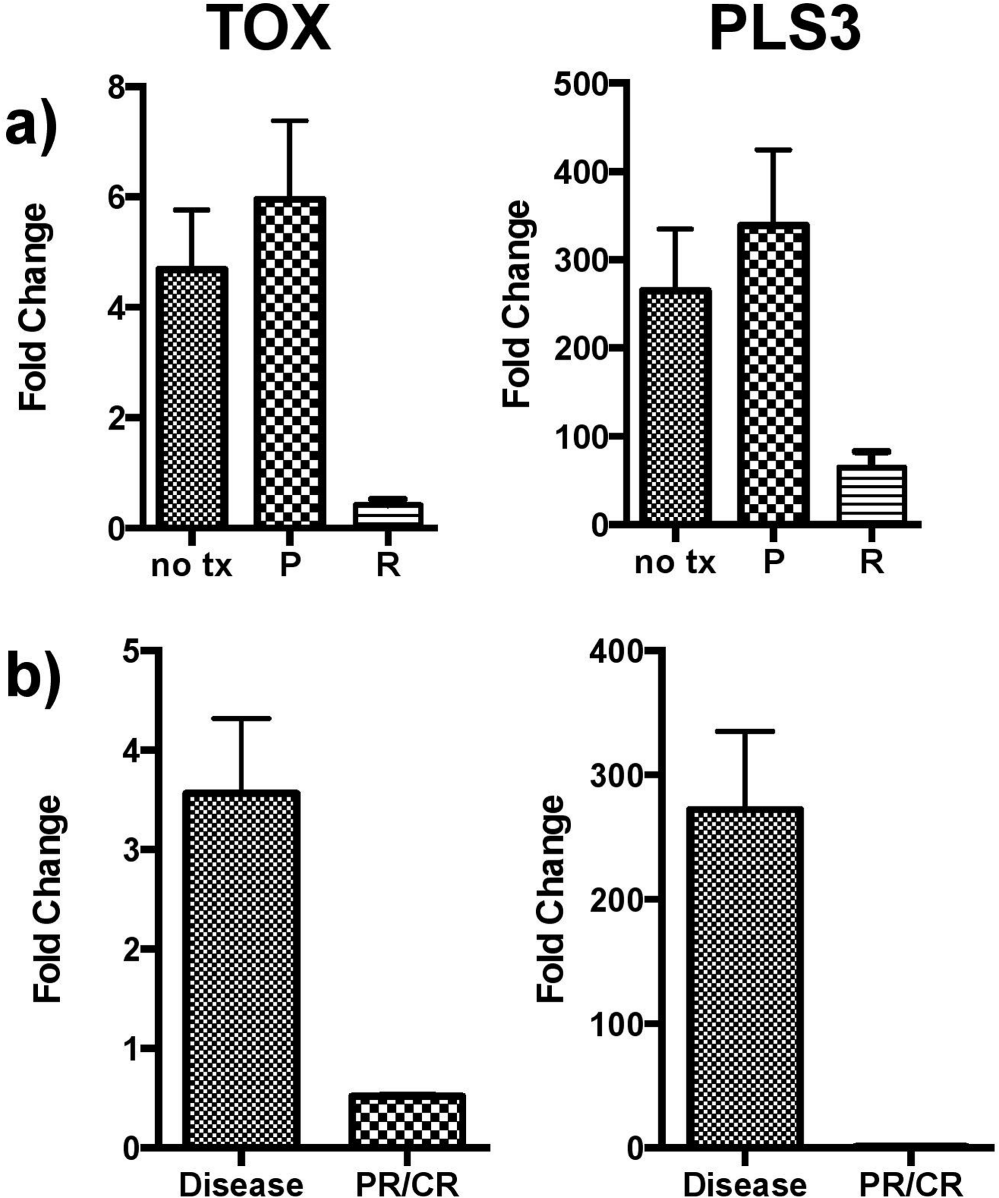
*TOX* and *PLS3* expression following drug treatment **a.** Effects of therapy on gene expression *in vitro*. Expression of both TOX and PLS3 in SS PBMCs normalizes with *in vitro* romidepsin (R) treatment but not pralatrexate (P) following 48-hour culture. Control samples (no tx) were patient PBMCs cultured in media with no drug for 48 hours. **b.** Gene expression changes *in vivo*. *TOX* and *PLS3* expression also normalized in CD4+ T-cells isolated from patients with partial or complete disease resolution following treatment with romidepsin. All pooled data is shown as mean +/− SEM.

To characterize the effects of treatment of CTCL on gene expression, cells isolated from three CTCL patients with treatment-naïve disease were treated in culture. Romidepsin, a histone deacetylase inhibitor, and pralatrexate, an antifolate analogue were selected for study. PBMCs isolated from SS patients were treated in culture with either romidepsin (10nM, 100nM, and 4 μM) or pralatrexate (5 μM and 20 μM) for 48 hours. Gene expression analysis was then conducted using RNA isolated from treated samples. Analysis revealed that *TOX* and *PLS3* expression normalizes with romidepsin treatment but not pralatrexate in CTCL PBMCs cultured for 48 hours (Figure 3a). In normal cells, a global decrease in gene expression was noted in response to treatment that was not specific to either drug (data not shown). Further, CD4+ T-cells were banked from two patients with active disease and from the same two patients following treatment with romidepsin and partial or complete resolution of disease. qRT-PCR analysis of these samples showed *TOX* and *PLS3* expression also normalize in CD4+ T-cells isolated from patients with disease resolution (Figure 3b).

### The TOX-RUNX3 pathway is dysregulated in SS

**Figure 4:**
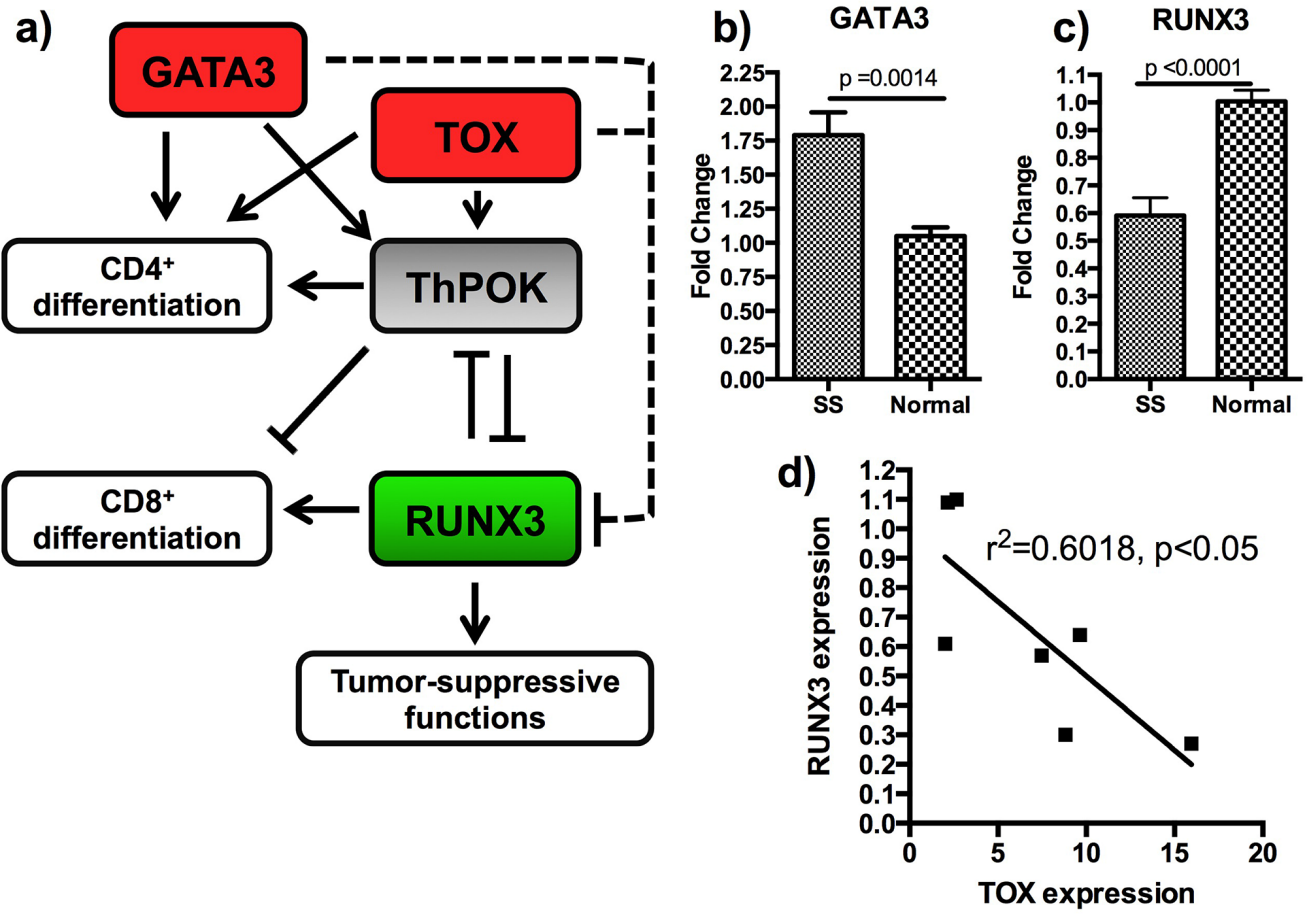
Additional members of the TOX-RUNX3 pathway are dysgregulated **a.** The TOX-RUNX3 pathway is show with genes differentially expressed following qRT-PCR analysis shown in red (upregulated) and green (downregulated.) The dashed lines represent indirect interactions that could include another intermediary, microRNA, or alteration of the RUNX3 promoter site. **b.** GATA3 is upregulated in SS CD4+ T-cells compared with healthy controls. **c.** RUNX3 is downregulated in SS CD4+ T-cells compared with healthy controls. **d.** RUNX3 expression is significantly inversely correlated to TOX expression. All pooled data is shown as mean +\− SEM.

Because TOX is a transcription factor, we sought to examine expression of its known downstream targets, particularly *RUNX3*, a tumor suppressor gene frequently deleted or transcriptionally silenced in cancer [21, 22]. TOX knockdown was recently shown to decrease the expression of ThPOK/ZBTB7B, a transcriptional regulator which serves as an intermediary in the TOX-RUNX3 pathway [23] and is also activated by GATA3 [24]. This pathway is involved in CD4/CD8 fate selection in T-cell development [16, 23], and *RUNX3* suppression is associated with Th2 skewing [25], which is frequently observed in patients with MF and is associated with advanced stage of disease. TOX was also recently shown to be associated with increased cell viability in malignant Sézary cells [26]. Our sequence-based transcriptome analysis indicated that *RUNX3* is downregulated in CD4+ cells from SS patients as compared with those from normal controls by a factor of −4.77, suggesting that the *TOX*→(+)*Intermediary*→ (-)*RUNX3* pathway may be important to SS pathogenesis (Figure 4a). qRT-PCR confirmed overexpression of GATA3 in SS CD4+ T-cells relative to healthy blood donor samples (Figure 4b), underexpression of RUNX3 (Figure 4c), and unchanged expression of ThPOK/ZBTB7B (data not shown), in addition to an inverse relationship between expression of TOX and RUNX3 in SS CD4+ T-cells (Figure 4d). Importantly, TOX has been hypothesized to play a role in PLS3 upregulation in SS [11]; however, our data did not demonstrate any significant correlation between TOX and PLS3 expression (not shown).

**Figure 5:**
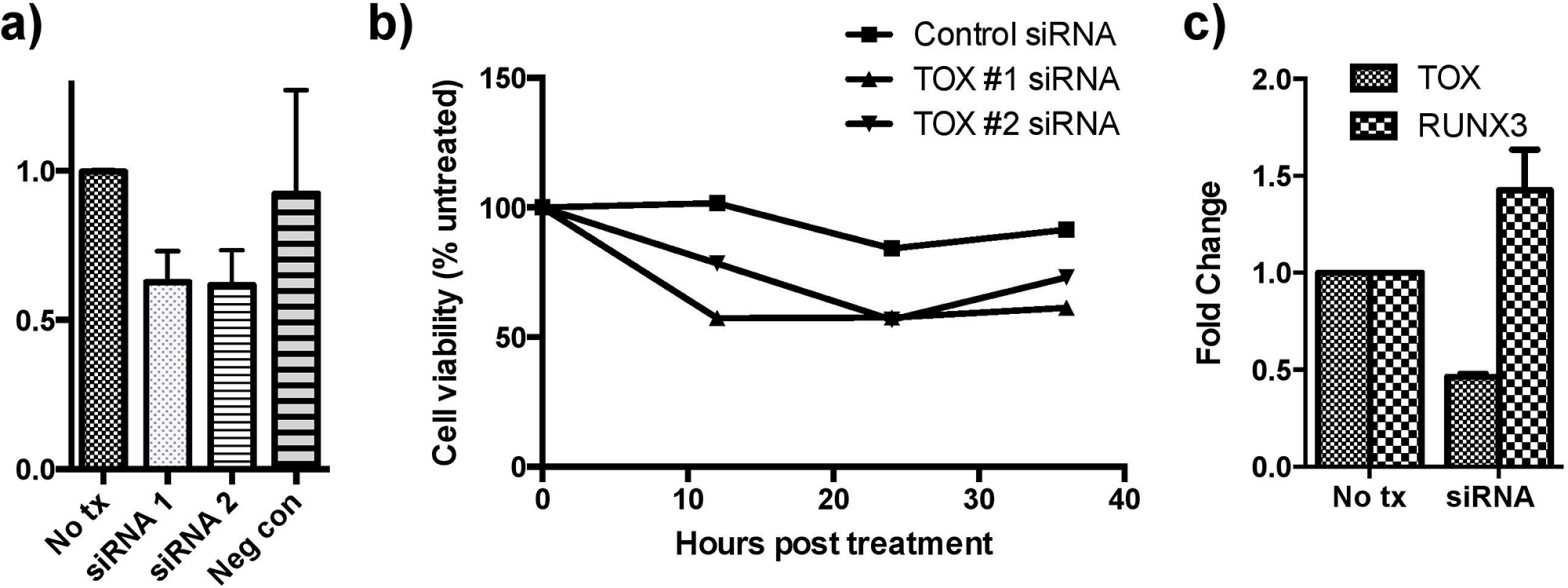
siRNA knockdown of TOX reduces cell viability and increases RUNX3 expression **a.** TOX siRNA knockdown was carried out using two constructs and confirmed with qRT-PCR analysis. **b.** TOX knockdown decreases cell viability more than negative control knockdown determined using a luminescence assay. 100% represents the viability of the untreated group at each respective time point. **c.** TOX knockdown also produced increased expression of RUNX3 based on qRT-PCR analysis. All pooled data is shown as mean +\− SEM.

Knockdown of TOX expression was performed using two different siRNA constructs (Figure 5a). Cell viability was studied following TOX siRNA knockdown with a resultant decrease in viability following treatment compared with cells treated with negative control siRNA and untreated cells (Figure 5b). Moreover, RUNX3 expression in cells treated with TOX siRNA was measured and was rescued following siRNA treatment (Figure 5c).

## DISCUSSION

*TOX* or thymocyte selection-associated high mobility group box is a transcription factor that contains an HMG-box DNA binding domain function. *TOX* is necessary for the development of CD4+ cell lineages and lymphoid tissue, but is not typically expressed in mature circulating CD4+ cells. [16] *TOX* indirectly suppresses *RUNX3*, a tumor suppressor implicated in many cancers, including solid organ malignancies, leukemia, and primary central nervous system lymphomas [21, 27, 28]. RUNX3 modulates the strength of Wnt signaling by complexing with β-catenin and T-Cell Factor 4 [22]. Our data, including transcriptome analysis and qRT-PCR results demonstrate that *TOX* is upregulated and *RUNX3* is downregulated in SS patients. Moreover, knockdown of TOX was associated with increase in *RUNX3* expression levels, indicating that TOX may act to decrease RUNX3′s tumor suppressor functions in SS. Importantly, TOX knockdown was associated with decreased cell viability, suggesting that it may play an important role in maintaining the malignant phenotype. Further studies on the effects of TOX expression on RUNX3 and cellular function included evasion of apoptosis and cell migration are warranted.

We examined regulation of RUNX3 by TOX in malignant CD4+ cells derived from PMBCs of Sézary syndrome patients. Interestingly, both TOX and GATA3, which was also previously demonstrated to be overexpressed in CTCL [11], are the negative regulators of RUNX3 expression. GATA3 and TOX proteins may exert their action on RUNX3 through epigenetic or posttranslational mechanisms, because a known intermediary, ThPOK, was not changed in our studies. In support of our conclusions, a ThPOK-independent mechanism of GATA3 effects on RUNX3 was recently reported [29]. McGirt *et al.* suggests that TOX itself may be upregulated following downregulation of microRNA-223, which is underexpressed in MF and has a binding site in the 3′UTR region of TOX [30]. Huang *et al.* recently demonstrated that TOX levels correlate with disease-specific mortality in SS patients, an observation that suggests the *in vitro* results achieved here are correlated to clinical pathogenesis and outcomes [26]. In light of the recent findings by our group and others, the role this pathway plays in pathogenesis of SS is an important area of continued study.

It has been previously suggested that *TOX* may upregulate *PLS3* in SS patients [11]. The overexpression of *PLS3* in SS peripheral blood mononuclear cells and SS CD4+ cells has been a single consistently reported finding through many studies [7–9]. In our next-generation sequence analysis, *PLS3* was significantly upregulated in purified malignant CD4+ cells from Sézary patients (83 times in SS compared to both MF patients and normal controls). Such significant and consistent upregulation of a gene not normally expressed in lymphocytes is suggestive of its pathological role in SS. In fact, PLS3 has been implicated in increasing resistance to apoptosis and SS cell migration toward chemokines [31]. However, we did not find any correlation between *TOX* and *PLS3* expression levels, and our data does not support a relationship between *TOX* and *PLS3*.

There are no markers currently available for definitive diagnosis of SS and MF. The diagnosis is based on a constellation of morphological, histological, and immunochemical parameters together with clinic-pathological correlation. Diagnosis of MF and SS is especially difficult in early stages of the diseases. We hypothesized that overexpression of PLS3 and TOX may result in high and specific expression of corresponding proteins that could be used diagnostically. To evaluate TOX and PLS3 diagnostic markers, we performed IHC staining of routine paraffin embedded MF and SS biopsy specimens. Using psoriasis samples as negative control, we observed specific positive staining of TOX and PLS3 in SS and MF. Importantly, we have previously demonstrated that TOX and CD4+ stain the same cells in CTCL samples using immunofluorescence [32]. Taken together, our observations indicate that TOX and PLS3 are strong candidates for future prospective biomarker validation studies.

In summary, we have described a novel pathway, the TOX-RUNX3 pathway, in SS and have demonstrated an inverse relationship between *TOX,* an established marker in CTCL, and a tumor suppressor gene *RUNX3*, suggestive of an important role of this pathway in disease pathogenesis. Further functional studies are necessary to confirm the role of each individual component of this pathway, including potential role of *GATA3*. Our results both contribute to the growing body of data defining multicomponent pathways important in disease etiology and pathogenesis, and discovering novel markers for diagnosis and potential therapeutic targets.

## MATERIALS AND METHODS

### Transcriptome analysis

Transcriptome data was analyzed from two sources. A single patient’s malignant CD4+ cells were compared to the same patient’s non-malignant CD4+ cells as we have previously described. [17] Next generation sequenced-based transcriptome analysis was conducted comparing both CD4+ T-cells from three SS patients and MF skin samples with CD4+ T-cells from three normal controls as we have previously described [18]. These data were analyzed using Ingenuity Pathways Analysis (IPA, http://www.ingenuity.com) and Metacore (Thomson Reuters, http://thomsonreuters.com) to determine oncogenic potential of upregulated genes.

### Cell sample collection, purification, and RNA and protein isolation

For validation of gene expression as well as drug and cell viability studies, peripheral blood was collected from nine histologically confirmed SS patients and four normal controls in total. Using Ficoll-Paque™ gradient centrifugation (Amersham Biosciences Corp, Piscataway, NF), peripheral blood mononuclear cells (PBMCs) were selected. The CD4+ cells were then isolated from whole PBMCs using MACS MicroBeads magnetic beads separation (Miltenyi Biotec, Inc., Auburn, CA). Purity of the resulting population of cells was checked with flow cytometry. From the CD4+ cells, RNA and protein were isolated using the RNeasy kit (Qiagen, Valencia, CA). For cDNA synthesis, total RNA (2 μg) was used for Reverse Transcription (RT) with Superscript II reverse transcriptase (Invitrogen, Karlsruhe, Germany) using oligo dT primers according to the recommendations of the manufacturer. The obtained cDNA was diluted 1:10 with ddH2O and 1 μl was used for each PCR reaction.

### qRT-PCR

Quantitative reverse transcription PCR was performed using TaqMan PCR master mix (Applied Biosystems, Grand Island, NY) together with TaqMan probes and primers (Applied Biosystems) using standard conditions. Human *B2M* was used as an internal control. The experiments were performed on a StepOnePlus™ Real-Time PCR System (Applied Biosystems). The expression levels of the studied genes were normalized to B2M expression. Experiments were carried out three separate days in triplicate. Each assay included a negative control that was lacking template DNA. Relative expression of RNAs was calculated using the two Δ*C*_T_ method [33]. Statistical analysis was performed using the student’s *t*-test.

### Immunohistochemistry

Histologically confirmed, formalin fixed paraffin embedded (FFPE) MF (*n* = 4) and SS (*n* = 2) tumor biopsies collected from our institutional tissue bank in accordance with an IRB-approved protocol were used for immunohistochemical analysis of candidate CTCL biomarkers, with FFPE psoriasis (*n* = 9) lesions selected for use as negative controls. Human esophagus and small intestine were used as positive controls for PLS3 and TOX respectively. Commercial antibodies were acquired for PLS3 (dilution 1:200, Abcam, Cambridge, MA) and TOX (dilution 1:250, Sigma Aldrich, St. Louis, MO). After deparaffinization, heat mediated epitope retrieval was conducted for 10 minutes (PLS3) and 60 minutes (TOX), respectively. Blocking was carried out with Super Block (Scy Tek, West Logan, UT) for PLS3 samples and Background Sniper (Biocare Medical, Concord, CA) for TOX. PLS3 slides were incubated with primary antibodies for 1 hour, biotinylated goat antirabbit IgG antibody (Scy Tek) for 20 minutes and AEC Substrate-chromogen (Scy Tek). TOX slides were incubated with primary antibodies for 1 hour, biotinylated goat antirabbit IgG antibody streptavidin/HRP (Biocare Medical, Concord, CA) for 45 minutes and Warp Red Chromagen for 15 minutes (Biocare Medica). Counterstaining was performed with Harris Hematoxylin for 15 seconds.

### Treatment with romidepsin and pralatrexate

To study the effects of treatment on gene transcription, two strategies were used. First, patient PBMCs from patients with active CTCL were collected as above. PBMCs were then cultured overnight in RPMI 1640 medium supplemented with interleukin-2 (BD Biosciences, San Jose, CA) 10 U/ml and interleukin-7 (BD Biosciences, San Jose, CA) 10 ng/ml both in a humidified incubator with 5% CO2 at 37°C and the next day divided into treatment groups: 4 μM, 100 nM, and 10 nM romidepsin and 20 μM and 5 μM pralatrexate. Romidepsin and pralatrexate were provided by the UPMC pharmacy. Romidepsin was dissolved in diluent composed of 80% propylene glycol and 20% dehydrated alcohol to a stock concentration of 2 mM and stored at −20°C. Dilutions of 100 and 10uM were made in RPMI 1640 and stored at −20°C for no more than 2 months. Pralatrexate was stored at a concentration of 20 mg/mL in −20°C at for no more than two months and was diluted as needed in RPMI 1640; these dilutions were used within one week. Each treatment was added 24 hours following culture initiation and carried out for 48 hours. Following treatment and prior to gene expression analysis, we performed flow cytometry to ensure that CD4+ cells comprised a similar percentage of each sample, ensuring that subsequent changes in gene expression were not the result of cell death or and altered population.

As described, RNA was isolated with RNAprotect and RNeasy products (Qiagen). Additionally, patient samples were banked from the same patients during active disease and after partial or complete clinical clearance of disease with romidepsin treatment. CD4+ cells were isolated from these samples using MACS magnetic beads (Miltenyi) and RNA was isolated as described. Finally, qRT-PCR was performed using the Quantitect reverse transcription kit (Qiagen) and TaqMan primers and PCR master mix (Applied Biosystems) on all samples. Relative expression of RNAs was calculated using the two Δ*C*_T_ method [33]. Statistical analysis was performed using the student’s *t*-test.

### Gene knockdown

To study the effects of TOX knockdown, siRNA knockdown was used. Two Silencer^®^ Select siRNA constructs (IDs s18841 and s18840, Ambion, Grand Island, NY) were transfected using *Trans*IT-siQUEST^®^ Transfection Reagent (Mirus Bio, Madison, WI) according to manufacturer’s instructions. Freshly harvested SS PBMCs were plated in a 24-well plate and 0.5 μl of the transfection reagent was added to 50 μl of Opti-MEM I Reduced-Serum Medium (life technologies, Grand Island, NY) then to each well. siRNA was added to a final concentration of 10nM. Incubation was carried out for 24–36 hours. Knockdown was confirmed with qRT-PCR as described above, and expression of RUNX3 was determined.

### Cell viability

Cells were cultured overnight in a 96-well microplate and the next day divided into four groups: untreated, TOX siRNA construct #1, TOX siRNA construct #2, and negative control siRNA. Each treatment was added 24 hours following culture initiation. At 24 hours post-treatment, a CellTiter-Glo^®^ Luminescent Cell Viability Assay (Promega, Madison, WI) was conducted according to manufacturer’s instructions. Plates were read using the Lmax Microplate Luminometer (Molecular Devices, Sunnyvale, CA) and SoftMax Pro Microplate Data Acquisition & Analysis Software (Molecular Devices). Each experiment was performed in quintuplicate, and statistical analysis comparing viability between treatment groups was conducted using the student’s *t*-test.

## SUPPLEMENTARY MATERIALS TABLE


