# Supplementary material for: Dysregulation of the TOX-RUNX3 pathway in cutaneous T-cell lymphoma
Source: Oncotarget. 2019 May 3;10(33):3104–13. doi: 10.18632/oncotarget.5742 (PMC6517103; doi:10.18632/oncotarget.5742)
Supplement: Supplementary file 1 [file oncotarget-10-3104-s001.pdf]

SUPPLEMENTARY TABLE

Patient	Age/Sex/ Race	Diagnosis, Stage
1	58 WM	T3N1M0B2/Stage IVA1, SS
2	80 WF	T3N0M0B2/Stage IVA1, SS
3	42 AAF	T3N1M0B2/Stage IVA1, SS
4	67 WF	T3N0M0B2/Stage IVA1, SS
5	67 WM	T3N0M0B2/Stage IVA1, SS

Patient characteristics for the five SS patient samples that were used to determine gene expression levels with qRT-PCR analysis.
